# Up-regulation of microRNA-375 ameliorates the damage of dopaminergic neurons, reduces oxidative stress and inflammation in Parkinson’s disease by inhibiting SP1

**DOI:** 10.18632/aging.102649

**Published:** 2020-01-11

**Authors:** Li-Jun Cai, Li Tu, Tian Li, Xiu-Lin Yang, Yi-Pin Ren, Ran Gu, Qian Zhang, Huan Yao, Xiang Qu, Qian Wang, Jin-Yong Tian

**Affiliations:** 1 Department of Neurology, The Affiliated Hospital of Guizhou Medical University, Guiyang 550004, PR. China; 2 Department of General Medical, The Affiliated Hospital of Guizhou Medical University, Guiyang 550004, PR. China; 3 Zunyi Medical University, Zunyi 563000, PR. China; 4 Department of Emergency, Guizhou Provincial People’s Hospital, Guiyang 550004, PR. China; 5 Department of Neurology, Guizhou Provincial People’s Hospital, Guiyang 550004, PR. China

**Keywords:** Parkinson's disease, microRNA-375, specificity protein 1, neurons, oxidative stress

## Abstract

Background: This study is conducted to investigate the protective role of elevated microRNA-375 (miR-375) in dopaminergic neurons in Parkinson’s disease through down-regulating transcription factor specificity protein 1 (SP1).

Results: The successfully modeled rats with Parkinson’s disease showed aggregated neurobehavioral change, increased neuroinflammatory response and oxidative stress, and lowered dopamine content. Parkinson’s disease rats treated with overexpressed miR-375 displayed improved neurobehavioral change, ameliorated neuroinflammatory response and oxidative stress, heightened dopamine content and abated neuronal apoptosis by down-regulating SP1. Up-regulation of SP1 reversed the protective effect of upregulated miR-375 on Parkinson’s disease.

Conclusion: Up-regulation of miR-375 ameliorated the damage of dopaminergic neurons, reduced oxidative stress and inflammation in Parkinson’s disease by inhibiting SP1.

Methods: Parkinson’s disease rat model was established by targeted injection of 6-hydroxydopamine to damage the substantia nigra striatum. The successfully modeled Parkinson’s disease rats were intracerebroventricularly injected with miR-375 mimics or pcDNA3.1-SP1. The functions of miR-375 and SP1 in neurobehavioral change, neuroinflammatory response, oxidative stress, dopamine content and expression of apoptosis-related proteins in the substantia nigra of Parkinson’s disease rats were evaluated. The target relation of miR-375 and SP1 was confirmed by bioinformatics analysis and dual luciferase reporter gene assay.

## INTRODUCTION

Parkinson’s disease is a neurodegenerative disorder mainly affecting the elderly. It is characterized by progressive damage to automatic behavior resulting in hypokinesia, sensory neglect and rigidity [1]. There are pathological signs in many brain regions, but the core symptoms of Parkinson’s disease are clearly related to the dopamine neuron degeneration and death in the substantia nigra pars compacta (SNpc) [2]. In recent years, the molecular basis of the death of dopamine neurons in Parkinson’s disease has been extensively discussed, such as oxidative stress, mitochondrial dysfunction, protein degradation dysregulation and potential neuroprotective treatments [3]. Significant progress has been made in parkinsonian neurodegeneration, which is primarily due to an in-depth understanding of the mechanisms of action of toxins, such as 6-hydroxydopamine (6-OHDA), rotenone, 1-methyl-4-phenyl-1,2,3,6-tetrahydropyridine, or others [4]. 6-OHDA is a neurotoxin that causes dopamine neuronal death and is commonly used to establish experimental models of Parkinson’s disease in rodents [5]. Although Parkinson’s disease has been investigated intensively for centuries, its etiology and pathogenesis remain unknown. In recent years, considerable evidence has demonstrated that microRNA (miRNA) deregulation is implicated in Parkinson’s disease [6–9].

MiRNAs are regarded as post-transcriptional gene expression regulators, which play essential roles in neuronal development, plasticity as well as diseases [10]. A study has suggested that miR-133b is important in the differentiation and maintenance of dopamine neurons, and is reduced in midbrain samples from Parkinson’s disease patients [11]. Besides, miR-205 has been demonstrated to be significantly down-regulated in Parkinson’s disease [12]. Firstly found in murine pancreatic β-cell line MIN6, microRNA-375 (miR-375) is a pancreatic islet-specific miRNA and controls glucose-induced insulin secretion [13]. Although miR-375 has been reported to be down-regulated in the development of mouse brain [14] and mouse dorsal root ganglia (DRG) following chronic morphine treatment [15], a clear role of miR-375 has not been defined in the development of Parkinson’s disease. Specificity protein 1 (SP1) is a transcription factor that is expressed in the brain, and its higher expression indicates a poorer neuronal survival [16]. As previously reported, elevated expression of SP1 was found in Alzheimer’s disease brains [17]. In addition, evidence has shown that SP1 was up-regulated in the progression of neurological diseases, such as Parkinson’s disease [18] and Alzheimer’s disease [19, 20]. Moreover, the target relation between miR-375 and SP1 has been previously identified [21, 22]. Based on aforementioned evidence, we hypothesized that the elevated miR-375 and inhibited SP1 might improve the 6-OHDA-induced damage of dopaminergic neurons in Parkinson’s disease.

## RESULTS

### Behavioral examination of rats with Parkinson’s disease in each group

The Parkinson’s disease rat models were established by injection of 6-OHDA in two points of the right medial forebrain bundle to explore the role of miR-375 and SP1 in pathological behaviors of 6-OHDA-induced Parkinson’s disease rats. Rotation test, open field test, stepping test and cylinder test were used to assess the pathological behaviors of Parkinson’s disease rats.

**Figure 1:**
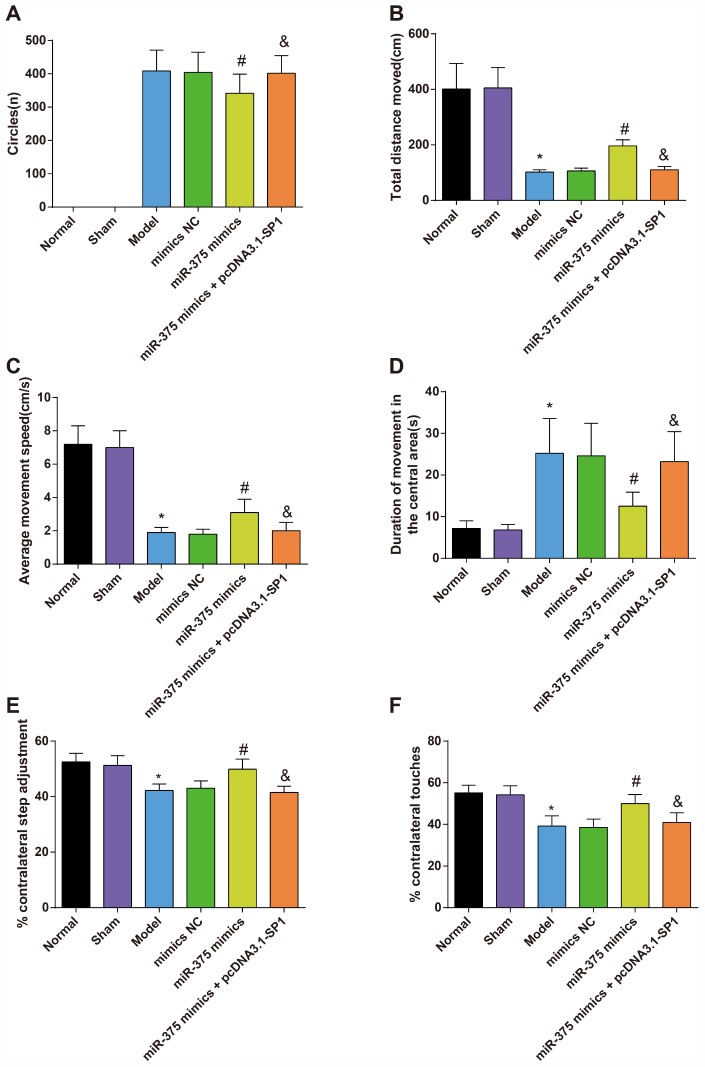
**The rotation test and open field test for rats in each group (n = 12).** (**A**) The number of rotation circles in each group of PD model rats induced by apomorphine; (**B**) The total distance moved of rats in each group; (**C**) The average movement speed of rats in each group; (**D**) The duration of movement in the central grid in one minute of rats in each group; (**E**) The results of the stepping test; (**F**) The results of the cylinder test; * *P* < 0.05 vs the normal group; # *P* < 0.05 vs the mimics NC group; & *P* < 0.05 vs the miR-375 mimics group. The measurement data were expressed as mean ± standard deviation and ANOVA was used for the comparison among multiple groups. After ANOVA analysis, the Tukey’s post-hoc test was used for pairwise comparison.

Results of the rotation test suggested that on the 25^th^ day after operation, none of the rats in the normal group and the sham group showed rotational behavior after induced by apomorphine, while the other four groups showed varying degrees of left rotation behaviors. The numbers of rotation circles of rats in the mimics NC and the miR-375 mimics + pcDNA3.1-SP1 group were not significantly different from that in the model group (*P* > 0.05). Compared with the mimics NC group, the number of rotation circles of rats in the miR-375 mimics group was significantly decreased (*P* < 0.05). The number of rotation circles in the miR-375 mimics + pcDNA3.1-SP1 group was significantly higher than that in the miR-375 mimics group (*P* < 0.05; Figure 1A).

Outcomes of the open field test reflected that there was no significant difference between the normal group and the sham group in the total distance moved, the average movement speed and duration of movement in the central grid in one minute of rats (all *P* > 0.05). Compared with the normal group, the total distance moved and the average movement speed decreased significantly while the duration of movement in the central grid in one minute increased significantly in the model group (all *P* < 0.05). However, there was no significant difference among the model group, the mimics NC group and the miR-375 mimics + pcDNA3.1-SP1 group in the total distance moved, the average movement speed and duration of movement in the central grid in one minute (all *P* > 0.05). In contrast to the mimics NC group, the total distance moved and the average movement speed increased significantly while the duration of movement in the central grid in one minute decreased significantly in the miR-375 mimics group (all *P* < 0.05). Relative to the miR-375 mimics group, the total distance moved and the average movement speed decreased significantly while the duration of movement in the central grid in one minute increased significantly in the miR-375 mimics + pcDNA3.1-SP1 group (all *P* < 0.05; Figure 1B–1D).

Results of stepping test (Figure 1E) and cylinder test (Figure 1F) mirrored that there was no considerable difference in the contralateral utilization ratio of rats between the normal group and the sham group (*P* > 0.05); the contralateral utilization ratio of 6-OHDA-induced rats was lower than that in the normal rats (*P* < 0.05); with the treatment of miR-375 mimics, the contralateral utilization ratio of rats was heightened (*P* < 0.05). Relative to the miR-375 mimics group, the contralateral utilization ratio of rats was declined in the miR-375 mimics + pcDNA3.1-SP1 group (*P* < 0.05).

The above results indicated that the up-regulation of miR-375 was able to improve the pathological behaviors of 6-OHDA-induced Parkinson’s disease rats

### Morphological observation of rats with Parkinson’s disease in each group

In order to observe the pathological injury in SNpc that caused by injection of 6-OHDA and to verify that whether the elevation of miR-375 could relieve pathological injury in SNpc that caused by injection of 6-OHDA, HE staining and Nissl staining were employed to observe the injury of cells in SNpc.

**Figure 2:**
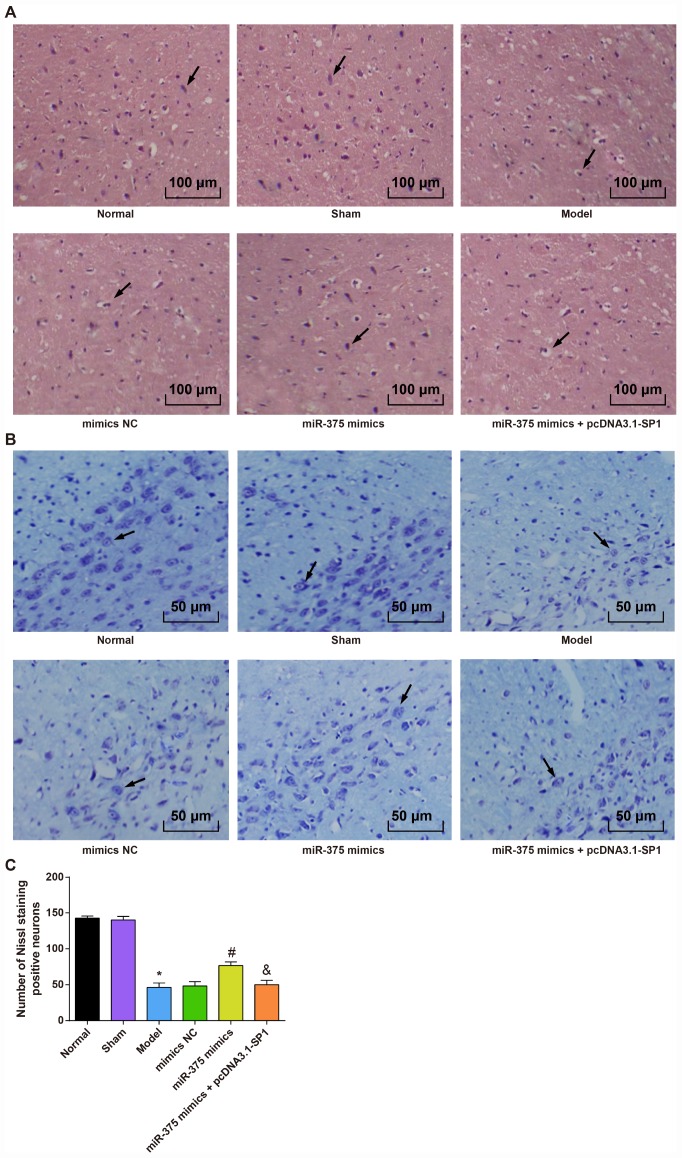
**Morphological observation of rats with PD in each group (n = 12).** (**A**) HE staining of substantia nigra in each group (× 100, the arrow indicates the neurons); (**B**) Nissl staining of substantia nigra in each group (× 200, the arrow indicates the Nissl positive neurons); (**C**) Comparison of the number of Neuron staining positive neurons in the substantia nigra of rats in each group; * *P* < 0.05 vs the normal group; # *P* < 0.05 vs the mimics NC group; & *P* < 0.05 vs the miR-375 mimics group. The measurement data were expressed as mean ± standard deviation and ANOVA was used for the comparison among multiple groups. After ANOVA analysis, the Tukey’s post-hoc test was used for pairwise comparison.

HE staining (Figure 2A) showed that cells in SNpc region were dense and arranged neatly in the normal group and the sham group, and cells in SNpc region of the model group and the miR-375 mimics + pcDNA3.1-SP1 group were significantly decreased with connective tissue proliferation. In the miR-375 mimics group, cells in SNpc region were dense, arranged in order and clear in interstitium.

Nissl staining (Figure 2B, 2C) showed that the numbers of Nissl positive neurons in SNpc region of rats in the normal group and the sham group were more than that in the model group (*P* < 0.05). There was no significant difference in the numbers of Nissl positive neurons in SNpc region among the model group, the mimics NC group and the miR-375 mimics + pcDNA3.1-SP1 group (all *P* > 0.05). The number of Nissl positive neurons in the miR-375 mimics group was significantly higher than that in the mimics NC group (*P* < 0.05), and the number of Nissl positive neurons in the miR-375 mimics + pcDNA3.1-SP1 group was significantly lower than that in the miR-375 mimics group (*P* < 0.05).

These outcomes revealed that the elevation of miR-375 has the ability to ameliorate the pathological injury in SNpc region of 6-OHDA-incuced Parkinson’s disease rats.

### Determination of dopamine in the striatum of rats with Parkinson’s disease in each group

**Figure 3:**
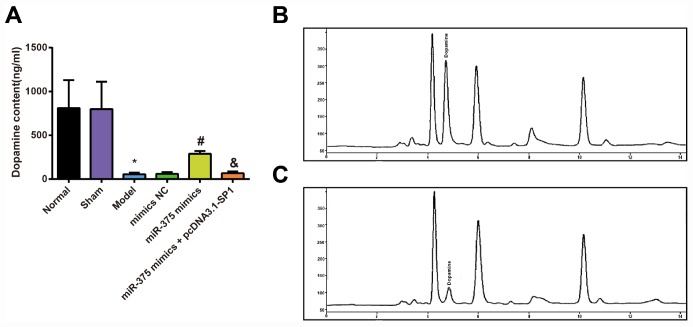
**Detection of dopamine content in striatum of rats in each group (n = 12).** (**A**) Dopamine content in the striatum of rats; (**B**) Dopamine content in the striatum of rats in the normal group was measured by HPLC; (**C**) Dopamine content in the striatum of rats in the model group was measured by HPLC; * *P* < 0.05 vs the normal group; # *P* < 0.05 vs the mimics NC group; & *P* < 0.05 vs the miR-375 mimics group. The measurement data were expressed as mean ± standard deviation and ANOVA was used for the comparison among multiple groups. After ANOVA analysis, the Tukey’s post-hoc test was used for pairwise comparison.

To assess the effects of 6-OHDA injection and elevated miR-375 on dopamine content of 6-OHDA-incuced Parkinson’s disease rats, HPLC was used to measure the dopamine content in the striatum of the rats. HPLC measurements of dopamine content in the striatum showed (Figure 3) that there was no significant difference in dopamine content in striatum of rats between the normal group and the sham group (*P* > 0.05). Compared with the normal group, dopamine content in striatum of rats was significantly decreased in the model group (*P* < 0.05). There was no significant difference in dopamine contents in striatum of rats among the model group, the mimics NC group and the miR-375 mimics + pcDNA3.1-SP1 group (all *P* > 0.05). The content of dopamine in striatum of rats in the miR-375 mimics group was significantly higher than that in the mimics NC group (*P* < 0.05), and the dopamine content in striatum of rats in the miR-375 mimics + pcDNA3.1-SP1 group was significantly lower than that in the miR-375 mimics group (*P* < 0.05). These data unearthed that the up-regulated miR-375 could reverse the decrease of dopamine content in the striatum of rats that induced by 6-OHDA treatment.

### TH positive neurons in substantia nigra of rats in each group

In order to evaluate the impacts of 6-OHDA injection and elevated miR-375 on neuronal injury in the SNpc region of 6-OHDA-incuced Parkinson’s disease rats, immunohistochemical staining was applied to assess the neuronal injury in the SNpc region of rats.

**Figure 4:**
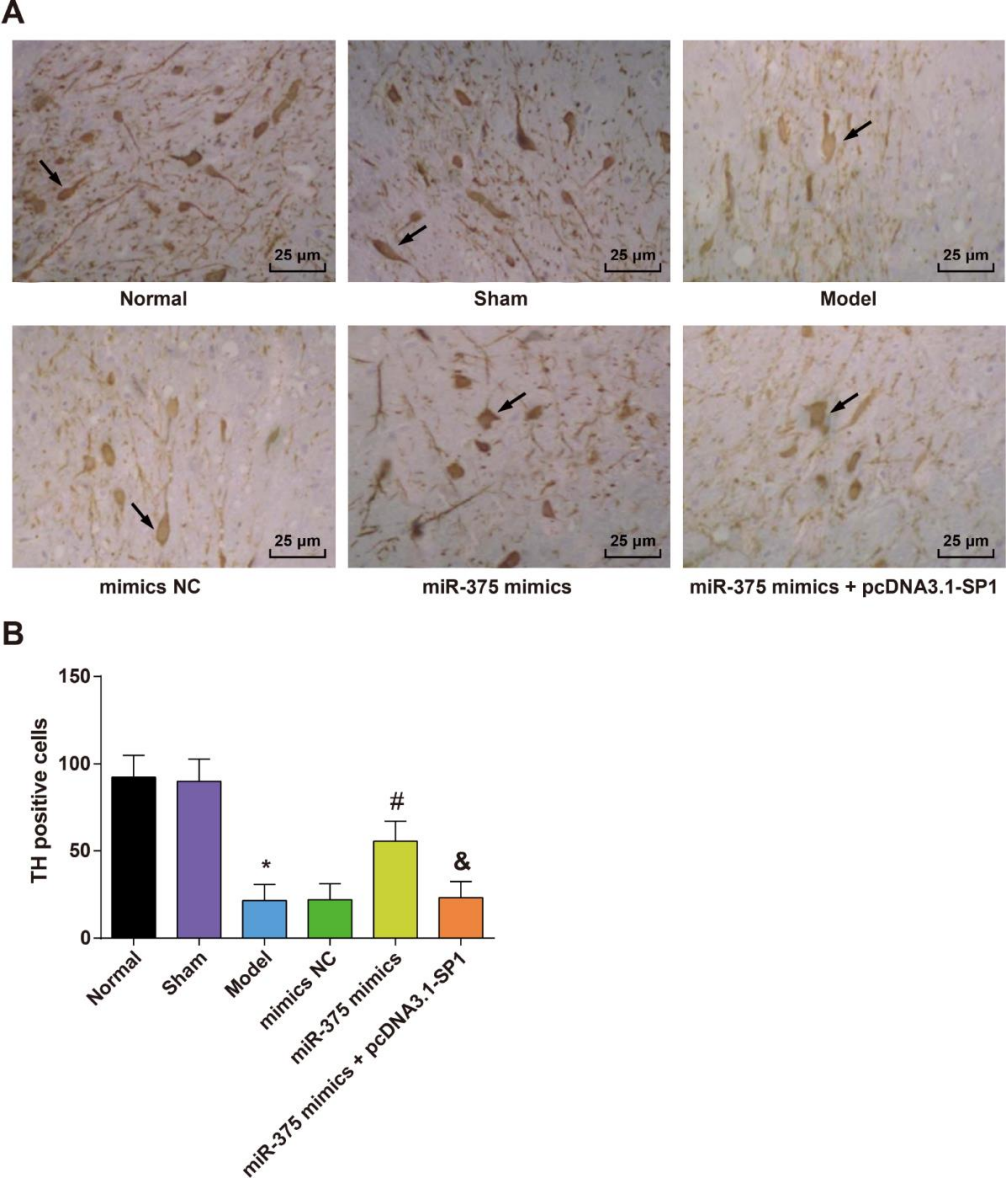
**Expression of TH positive neurons in the substantia nigra of rats in each group (n = 12).** (**A**) Immunohistochemical staining of TH in substantia nigra of rats in each group (× 400, the arrow indicates the TH positive neurons); (**B**) Number of TH positive cells in substantia nigra of rats in each group; * *P* < 0.05 vs the normal group; # *P* < 0.05 vs the mimics NC group; & *P* < 0.05 vs the miR-375 mimics group. The measurement data were expressed as mean ± standard deviation and ANOVA was used for the comparison among multiple groups. After ANOVA analysis, the Tukey’s post-hoc test was used for pairwise comparison.

Immunohistochemical detection of TH in the substantia nigra of rats in each group (Figure 4A) showed that the TH immunoreactive neurons in the SNpc region were brown, which were densely distributed, large in number and cell bodies, with obvious neuronal protuberance of rats in the normal and sham groups. In the model group, the number of TH immunoreactive neurons in the SNpc region was significantly decreased, and the cell body contours and processes of the neurons were not clear. In the miR-375 mimics group, the TH immunoreactive neurons in the SNpc area were brown, which were stained deeply, and the number of TH immunoreactive neurons was larger. More neuronal processes could be seen in the SNpc region. The number of TH positive cells in the substantia nigra of rats in each group is shown in Figure 4B. The results suggested that the number of TH positive cells in substantia nigra in the model group was significantly lower than that in the normal group and the sham group (*P* < 0.05). The number of TH positive neurons in the miR-375 mimics group was significantly higher than that in the model group and the mimics NC group (*P* < 0.05). There was no significant difference in the number of TH positive neurons among the model group, the mimics NC group and the miR-375 mimics + pcDNA3.1-SP1 group (*P* > 0.05). The number of TH positive neurons in the miR-375 mimics + pcDNA3.1-SP1 group was significantly less than that in the miR-375 mimics group (*P* < 0.05). We could find from the outcomes that the elevation of miR-375 was able to reverse the decrease of TH positive neurons that induced by 6-OHDA treatment.

### Expression of apoptosis-related proteins Caspase-3, Bax and Bcl-2 in substantia nigra of rats in each group

The protein expression levels of apoptosis-related proteins (Caspase-3, Bax and Bcl-2) were determined by Western blot analysis to measure the effects of 6-OHDA injection and elevated miR-375 on cell apoptosis in substantia nigra.

**Figure 5:**
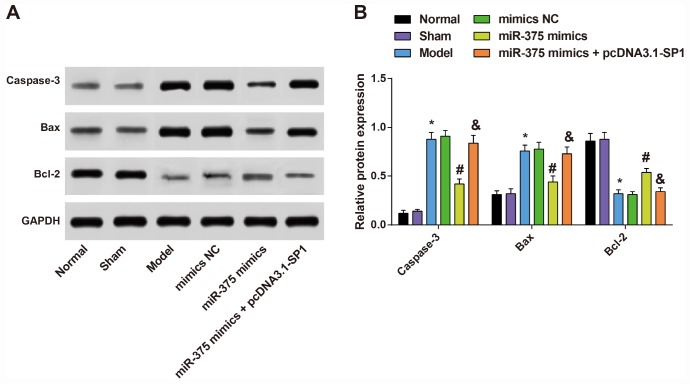
**Expression of apoptosis-related proteins Caspase-3, Bax and Bcl-2 in substantia nigra of rats in each group (n = 12).** (**A**) Protein bands of apoptosis-related proteins Caspase-3, Bax and Bcl-2 in substantia nigra of rats in each group; (**B**) Expression of apoptosis-related proteins Caspase-3, Bax and Bcl-2 in substantia nigra of rats in each group; * *P* < 0.05 vs the normal group; # *P* < 0.05 vs the mimics NC group; & *P* < 0.05 vs the miR-375 mimics group. The measurement data were expressed as mean ± standard deviation and ANOVA was used for the comparison among multiple groups. After ANOVA analysis, the Tukey’s post-hoc test was used for pairwise comparison.

As shown in Figure 5, the results showed that the expression of apoptosis-related proteins Caspase-3, Bax and Bcl-2 in substantia nigra of rats in the normal group and the sham group were not significantly different (all *P* > 0.05). In the model group, the protein expression of pro-apoptotic factors Caspase-3 and Bax was up-regulated, and the expression of anti-apoptotic factor Bcl-2 was down-regulated in the substantia nigra relative to the normal group (all *P* < 0.05). There was no significant difference in the expression of Caspase-3, Bax and Bcl-2 in the substantia nigra of rats among the model group, the mimics NC group and the miR-375 mimics + pcDNA3.1-SP1 group (all *P* > 0.05). The protein expression of Caspase-3 and Bax was down-regulated, and the expression of Bcl-2 was up-regulated in the substantia nigra of rats in the miR-375 mimics group relative to the mimics NC group (all *P* < 0.05). Compared with the miR-375 mimics group, the protein expression of Caspase-3 and Bax was up-regulated, and the expression of Bcl-2 was down-regulated in the substantia nigra of rats in the miR-375 mimics + pcDNA3.1-SP1 group (all *P* < 0.05). It could be found that the elevated miR-375 could reverse the promotion of cell apoptosis in substantia nigra of rats that induced by 6-OHDA treatment.

### Changes of oxidative stress in substantia nigra of rats in each group

**Figure 6:**
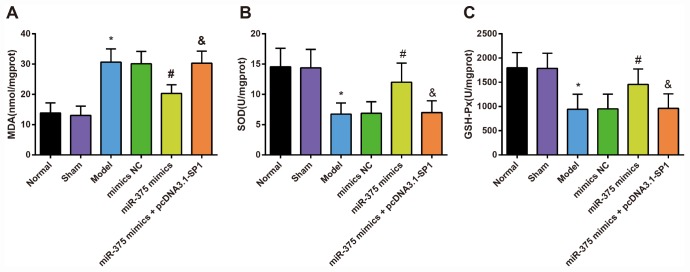
**Changes of MDA content, SOD and GSH-Px activity in substantia nigra of rats in each group (n = 12).** (**A**) MDA content in substantia nigra of rats in each group; (**B**) SOD activity in substantia nigra of rats in each group; (**C**) GSH-Px activity in substantia nigra of rats in each group; * *P* < 0.05 vs the normal group; # *P* < 0.05 vs the mimics NC group; & *P* < 0.05 vs the miR-375 mimics group. The measurement data were expressed as mean ± standard deviation and ANOVA was used for the comparison among multiple groups. After ANOVA analysis, the Tukey’s post-hoc test was used for pairwise comparison.

The contents of oxidative stress factors (SOD, GSH-Px and MDA) in the substantia nigra were determined by ELISA to assess the role of up-regulated miR-375 in oxidative stress response of 6-OHDA-induced Parkinson’s disease rats (Figure 6). The results indicated that there was no significant difference in the contents of SOD, GSH-Px and MDA in the substantia nigra between the normal group and the sham group (all *P* > 0.05). Compared with the normal group, the content of MDA increased significantly while the activities of SOD and GSH-Px decreased significantly in the substantia nigra of rats in the model group (all *P* < 0.05). There was no significant difference in the contents of SOD, GSH-Px and MDA in the substantia nigra among the model group, the mimics NC group and the miR-375 mimics + pcDNA3.1-SP1 group (all *P* > 0.05). The content of MDA decreased significantly while the activities of SOD and GSH-Px increased significantly in the substantia nigra of rats in the miR-375 mimics group relative to the mimics NC group (all *P* < 0.05). Compared with the miR-375 mimics group, the content of MDA was significantly increased, while the activities of SOD and GSH-Px were significantly decreased in substantia nigra of rats in the miR-375 mimics + pcDNA3.1-SP1 group (all *P* < 0.05). These results mirrored that the elevation of miR-375 was able to attenuate the oxidative stress response in the substantia nigra of rats that induced by 6-OHDA treatment.

### Expression of inflammatory factors TNF-α, IL-6 and IL-1β in substantia nigra of rats in each group

The mRNA expression and protein expression of inflammatory factors (TNF-α, IL-6 and IL-1β) in the substantia nigra were respectively measured by RT-qPCR and ELISA to assess the effect of elevated miR-375 in inflammatory response of 6-OHDA-induced Parkinson’s disease rats.

**Figure 7:**
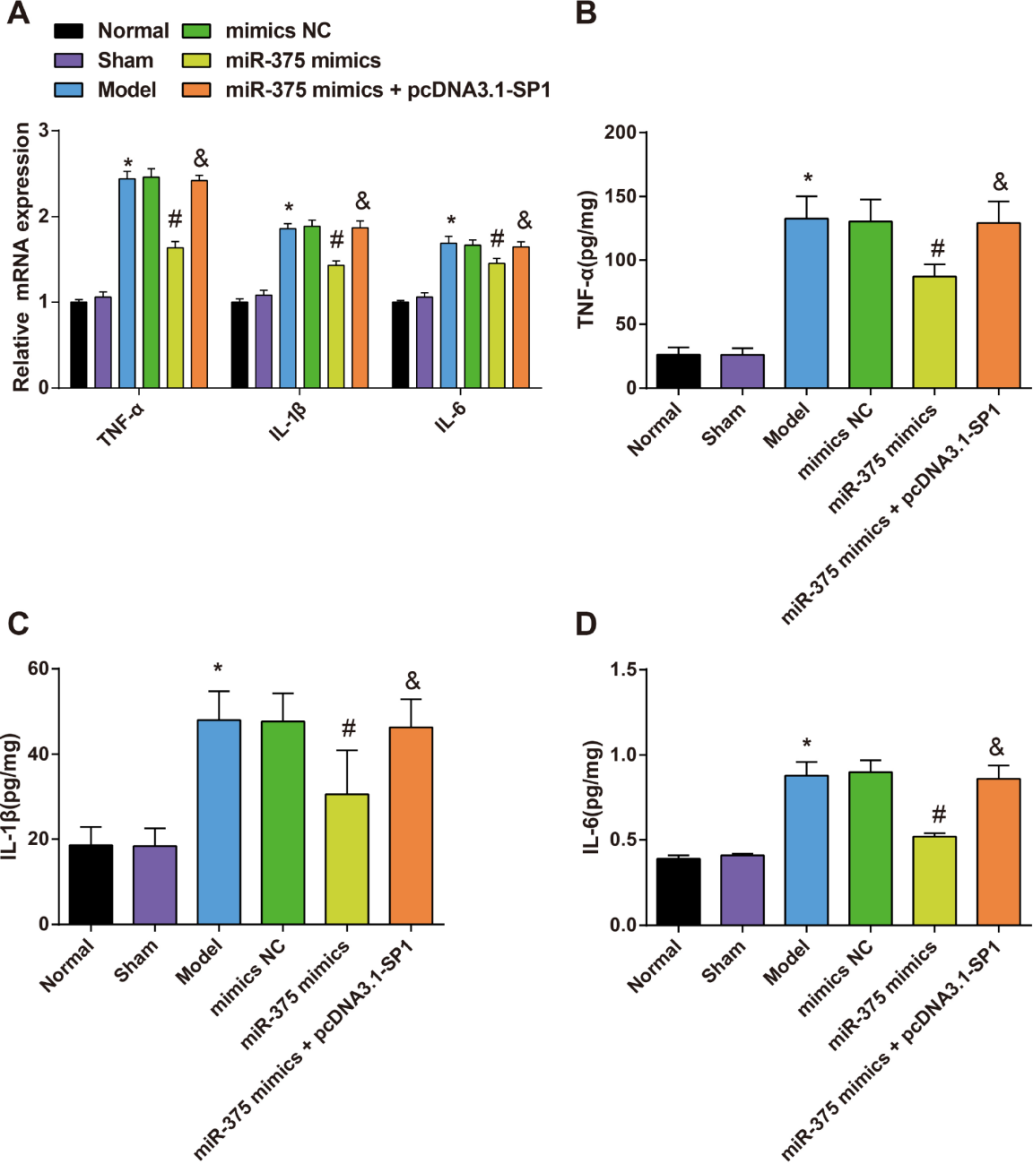
**Expression of inflammatory factors TNF-α, IL-1β and IL-6 in substantia nigra of rats in each group (n = 12).** (**A**) mRNA expression of inflammatory factors TNF-α, IL-1β and IL-6 in substantia nigra of rats in each group; (**B**–**D**) Expression of inflammatory factors TNF-α, IL-1β and IL-6 in substantia nigra of rats in each group; * *P* < 0.05 vs the normal group; # *P* < 0.05 vs the mimics NC group; & *P* < 0.05 vs the miR-375 mimics group. The measurement data were expressed as mean ± standard deviation and ANOVA was used for the comparison among multiple groups. After ANOVA analysis, the Tukey’s post-hoc test was used for pairwise comparison.

The mRNA expression of TNF-α, IL-6 and IL-1β in substantia nigra of rats in each group was detected by RT-qPCR and the protein expression was evaluated by ELISA (Figure 7). The results suggested that there was no significant difference in TNF-α, IL-6 and IL-1β mRNA and protein expression in the substantia nigra between the normal group and the sham group (all *P* > 0.05). Compared with the normal group, TNF-α, IL-6 and IL-1β mRNA and protein expression was increased significantly in the substantia nigra of rats in the model group (all *P* < 0.05). There was no significant difference in TNF-α, IL-6 and IL-1β mRNA and protein expression in the substantia nigra among the model group, the mimics NC group and the miR-375 mimics + pcDNA3.1-SP1 group (all *P* > 0.05). TNF-α, IL-6 and IL-1β mRNA and protein expression was decreased significantly in the substantia nigra of rats in the miR-375 mimics group relative to the mimics NC group (all *P* < 0.05). Compared with the miR-375 mimics group, TNF-α, IL-6 and IL-1β mRNA and protein expression was significantly increased in substantia nigra of rats in the miR-375 mimics + pcDNA3.1-SP1 group (all *P* < 0.05). The above outcomes indicated that the up-regulation of miR-375 has the capacity to relieve the inflammatory response in substantia nigra of the 6-OHDA-induced Parkinson’s disease rats.

### Expression of miR-375 and SP1 in the substantia nigra of rats in each group

To measure the impact of 6-OHDA injection on the expression of miR-375 and SP1 in the substantia nigra, and the regulatory role of miR-375 in SP1 expression, we used RT-qPCR and Western blot analysis to determine the expression of miR-375 and SP1 in the substantia nigra of rats in each group.

**Figure 8:**
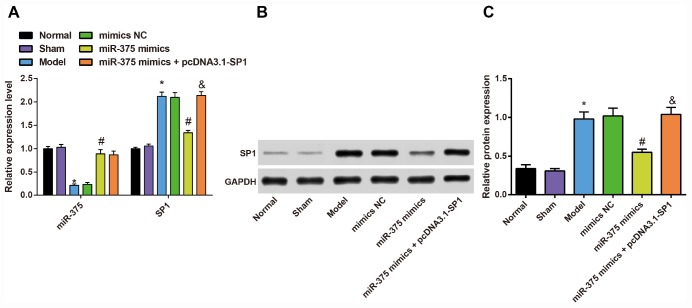
**Expression of miR-375 and SP1 in the substantia nigra of rats in each group (n = 12).** (**A**) Expression of miR-375 and SP1 mRNA in the substantia nigra of rats in each group; (**B**, **C**) Expression of SP1 protein in the substantia nigra of rats in each group; * *P* < 0.05 vs the normal group; # *P* < 0.05 vs the mimics NC group; & *P* < 0.05 vs the miR-375 mimics group. The measurement data were expressed as mean ± standard deviation and ANOVA was used for the comparison among multiple groups. After ANOVA analysis, the Tukey’s post-hoc test was used for pairwise comparison.

The results showed (Figure 8A) that the expression of miR-375 in substantia nigra of rats in the normal group and the sham group had no significant difference (*P* > 0.05). Compared with the normal group, the expression of miR-375 in the substantia nigra of rats in the model group was decreased (*P* < 0.05), but the expression of miR-375 in the substantia nigra of rats in the model group was not significantly different from that in the mimics NC group (*P* > 0.05). In comparison to the mimics NC group, the expression of miR-375 in substantia nigra of rats was significantly increased in the miR-375 mimics group (*P* < 0.05). There was no significant difference in the expression of miR-375 in substantia nigra of rats in the miR-375 mimics + pcDNA3.1-SP1 rats compared with the miR-375 mimics group (*P* > 0.05).

RT-qPCR and western blot analysis were used to detect the expression of SP1 mRNA and protein in substantia nigra of rats (Figure 8A–8C). The results showed that the expression of SP1 mRNA and protein in substantia nigra of rats in the normal group and the sham group had no significant difference (*P* > 0.05). Compared with the normal group, the expression of SP1 mRNA and protein was significantly increased in the substantia nigra of rats in the model group (*P* < 0.05), while the expression of SP1 mRNA and protein in the substantia nigra of rats was not significantly different in the model group, mimics NC group and the miR-375 mimics + pcDNA3.1-SP1 group (*P* > 0.05). In comparison to the mimics NC group, the expression of SP1 mRNA and protein in substantia nigra of rats was significantly decreased in the miR-375 mimics group (*P* < 0.05). The expression of SP1 mRNA and protein in substantia nigra of rats in the miR-375 mimics + pcDNA3.1-SP1 group was significantly higher than that in the miR-375 mimics group (*P* < 0.05).

These data reflected that miR-375 was poorly expressed, while SP1 was highly expressed in the substantia nigra of Parkinson’s disease rats, and the up-regulated miR-375 could inhibit the expression of SP1.

### SP1 is determined as a target gene of miR-375

**Figure 9:**
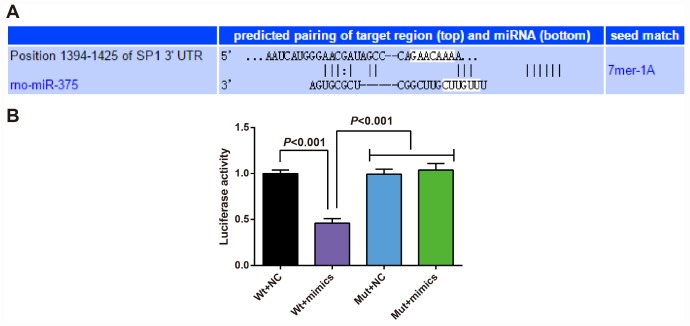
**SP1 is determined as a target gene of miR-375.** (**A**) TargetScan prediction of target site for SP1 and miR-375; (**B**) Detection of the luciferase activity by dual luciferase reporter gene assay; the independent experiment was repeated 3 times. The data represent the mean ± standard deviation of three independent experiments.

In order to analyze the regulatory relation between miR-375 and SP1, the binding sites of SP1 and miR-375 were determined by an online prediction software TargetScan. The sequence of 3'-UTR region combined with SP1 mRNA and miR-375 is shown in Figure 9A. In order to prove that the predicted binding sites of miR-375 resulted in the change of luciferase activity, the mutation type (MUT) sequence and wild type (WT) sequence of SP1 3'UTR with or without miR-375 were designed respectively to insert into the reporter plasmid. Using luciferase activity assay, the recombinant plasmids of miR-375 mimics, WT-miR-375/SP1 or MUT-miR-375/SP1 were cotransfected into 293T cells. The results showed that miR-375 mimics had no significant effect on luciferase activity in MUT-miR-375/SP1 plasmid, but significantly decreased luciferase activity in WT-miR-375/SP1 plasmid (Figure 9B), indicating that SP1 was targeted by miR-375.

## DISCUSSION

Parkinson’s disease commonly develops due to a complex net of pathological changes, such as oxidative stress, neuroinflammation and mitochondrial and proteasomal dysfunction [23]. It has been reported that the exposure of dopamine neurons to 6-OHDA contributes to the neurotoxin uptake by the dopamine transporter together with its subsequent oxidation to both reactive oxygen species and free radicals [5]. However, the deficiency in efficient biomarkers impedes timely intervention of Parkinson’s disease, which demands continuous endeavors [24]. Even though the 6-OHDA is commonly utilized to establishment experimental models of Parkinson’s disease, it remains unclear how 6-OHDA relates the disease mechanism in humans. In this present study, we aim to investigate the roles of miR-375 and the transcription factor SP1 in the protection of dopaminergic neurons in Parkinson’s disease. Altogether, the results in our study demonstrate that overexpression of miR-375 down-regulated the expression of SP1, thus improving the damage of dopaminergic neurons caused by 6-OHDA in Parkinson’s disease, and may play a protective role by reducing oxidative stress damage.

First of all, we found out that the expression of miR-375 was decreased in Parkinson’s disease. In recent years, genome-wide miRNA expression profiling studies elucidated that miR-375 is found to be significantly down-regulated in malignant tumors, such as hepatocellular carcinoma, gastric cancer and glioma [25–27]. Additionally, some recent studies have also identified the expression of miR-375 acts as a tumor suppressor in a large number of tissues [28, 29]. In addition, this study suggested that the overexpression of miR-375 ameliorated the damage of dopaminergic neurons, reduced oxidative stress and inflammation in Parkinson’s disease. There are some studies concentrated on the relationship between miR-375 and nervous system diseases. For instance, a study has suggested that miR-375 is elevated in the development of human spinal motor neuron, and its elevation can facilitate spinal motor neurogenesis [30]. Moreover, Li *et al*. have illuminated that the up-regulation of miR-375 could ameliorate morphine analgesic tolerance in mouse dorsal root ganglia [15], and it has been recently testified that the inhibition of miR-375 was able to attenuate ketamine-induced neurotoxicity in human embryonic stem cell derived neurons [31].

As for the relationship between SP1 and Parkinson’s disease, some important transcription factors that decide their phenotype were overexpressed in the dopamine neurons, and some of these transcription factors keeping their overexpression into adulthood [32]. In this present study, we also found that the expression of SP1 was increased in Parkinson’s disease progression. In addition, this study suggested that the down-regulation of SP1 ameliorated the damage of dopaminergic neurons, reduced oxidative stress and inflammation in Parkinson’s disease. A study has suggested that the elevation of SP1 levels could trigger cell death in neurodegenerative disorders, including Alzheimer’s disease [16], making it an interesting candidate for therapeutic intervention of neurodegenerative disorders. Except that, SP1 is dramatically up-regulated in embryonic cortical neurons in response to oxidative stress [33], and it up-regulates the expression of manganese superoxide dismutase, which protects the mitochondrion from reactive oxygen species-induced damage [34], suggesting SP1 might be a oxidative stress-induced transcription factor that represses neuronal survival in cortical neurons. Furthermore, inhibition of SP1 has been proved to delay neuron loss and prolong lifespan [35].

It has been demonstrated that miRNA deregulation contributes to neuronal dysfunction in neurodegenerative disorders, which underlies the altered expression of key target genes [36], and miRNAs are able to conduct targeted-mRNA degradation or translational inhibition via sequence complementarity to 3’UTR of mRNAs [37, 38]. This study further verified the target relationship between miR-375 and SP1. Similar to our results, miR-375 is reduced in squamous cervical cancer and the up-regulation of miR-375 inhibits cell migration and invasion through suppressing transcription factor SP1 [21]. Another study also confirmed that the overexpressed miR-375 could suppress the synthesis and secretion of catecholamines in rat adrenal medulla by reducing SP1 [22]. Moreover, elevated miR-375 is found to inhibit the invasion and metastasis, and regulate epithelial-mesenchymal transition-associated genes of colorectal cancer via inhibiting SP1 [39].

## CONCLUSION

In summary, our study suggests that the up-regulation of miR-375 ameliorated the damage of dopaminergic neurons, reduced oxidative stress and inflammation in Parkinson’s disease by inhibiting SP1. Thus, miR-375 and its target gene SP1 have potential as biomarkers and as therapeutic targets for Parkinson’s disease.

## MATERIALS AND METHODS

### Ethics statement

This study protocol was approved by the Experimental Animal Ethics Committee of Guizhou Provincial People’s Hospital. The animal experiment was strictly adhered to the principle to minimize the pain and suffering to experimental animals.

### Experimental animals

Healthy male Wistar rats, weighted 220 g-250 g and aged 3 months [40], were provided by Hunan SJA Laboratory Animal Co., Ltd (Hunan, China). The rats were kept in the room at 22-25°C with 12 h day/night cycle, and they had free access to water and food. The cages and pads were changed every 2 days, and the rats were adapted to the laboratory environment for one week before experiment.

### Establishment of rat models of Parkinson’s disease

The randomly-assigned experimental rats were anesthetized by 0.5 mL/100 g chloral hydrate (Shanghai Chemical Reagent Co. Ltd., Shanghai, China) and fixed in the prone position in a stereoscopic locator of the rat’s brain (RWD Life Science Co., Shenzhen, China). The head skin of rat was disinfected with 75% ethanol and cut open by a sharp surgical knife, thereby the skull was exposed and the anterior fontanelle was chosen as the origin point. Two points of the right medial forebrain tract were drilled according to the Paxinos & Watson pattern: (1) 4.0 mm behind the anterior fontanelle, 0.8 mm right of the anterior fontanelle and 0.8 mm below the endocranium; (2) 4.4 mm behind the anterior fontanelle, 1.2 mm right of the anterior fontanelle and 7.8 mm below the endocranium. The dosage of 6-OHDA (Sigma-Aldrich, St. Louis, MO, USA) was 2.25 μL and 2.7 μL (1 μL/min for 5 min). The needle was pulled out slowly, the skin was disinfected and the scalp was sutured. Penicillin was used to prevent infection for 3 days, with 200,000 U per day. The same method was used to inject 2.25 μL and 2.7 μL normal saline into the medial forebrain tract of rats in the sham group.

### Animal treatment

Seventy-two healthy male Wistar rats were randomly divided into 6 groups (n = 12) by random digital table: the normal group (without any treatment), the sham group (two points injection of right medial forebrain bundle of normal saline), the model group (two-point injection of the right medial forebrain bundle of 6-OHDA), the mimics negative control (NC) (the miR-375 mimics NC was used to pretreat the substantia nigra of rats, and then the two points injection of the right medial forebrain bundle of 6-OHDA was performed), the miR-375 mimics group (miR-375 mimics was used to pretreat the substantia nigra of rats, and then two points injection into the right medial forebrain bundle of 6-OHDA was performed) and the miR-375 mimics + pcDNA3.1-SP1 group (miR-375 mimics and pcDNA3.1-SP1 were used to pretreat the substantia nigra of rats, and then two points injection to the right medial forebrain bundle of 6-OHDA was performed). The nigra injection steps were the same with the model establishment procedures. The injection dose of mimics NC, miR-375 mimics and the combination of miR-375 mimics and pcDNA3.1-SP1 was 1 μL each and the speed was 0.1 μL/min for 10 min. The needle was pulled out slowly, the skin was disinfected and the scalp was sutured. Penicillin was used to prevent infection for 3 days, with 200,000 U per day. The mimics NC, miR-375 mimics and pcDNA3.1-SP1 were provided by Shanghai Genechem Co., Ltd. (Shanghai, China).

### Animal behavior determination

Rotation test: The rats of each model group and intervention group were intraperitoneally injected with apomorphine (0.5 mg/kg) on the 25^th^ day after 6-OHDA injection [41]. The rotational behavior of the rats was observed and recorded after 5 min. The standard was to rotate to the opposite side of the injury with the opposite hind limb as the supporting point, the head and tail were joined, and the body bent into a circle and rotated. The rotation times of each group of rats in 30 min were counted.

Open field test: The length, width and height of the open field test box were 100 × 100 × 40 cm, the bottom was divided into 25 squares of 20 × 20 cm, and the inner wall and bottom were painted black. The rats of each model group and the intervention group were placed in the center of the open field experiment box in turn, and the walking distance, average velocity and central lattice residence time of the rats were recorded in one minute. The open field test box was disinfected with 75% alcohol after each rat was tested, and the remaining odors were removed for the next test.

Stepping test: The rats were forced to support their bodies by the fore limbs on a desk (length at 10 cm and width at 40 cm) with their tails grasped by the experimenter and the fore limbs coated with edible pigment, then slowly dragged backward by the experimenter for 1 m, and the steps of the bilateral fore limbs were recorded. Before the experiment, the rats were stroked and grasped by the experimenter for 3 min to make it easier for the rats to adapt to the grasp. The contralateral step adjustment (%) = (number of contralateral steps/number of total steps) × 100%.

Cylinder test: The rats were put into a transparent cylinder (diameter at 21 cm and height at 30 cm) and the times of their left, right and bilateral fore limbs touching the wall in 5 min were recorded. The posterior limbs were set as the supporting points, and unilateral or bilateral fore limbs touching the wall then returning to the bottom was recorded as one time of contralateral touch (one side of the limb firstly touching wall and followed by the other side of the limb was recorded as a bilateral touch). The cylinder was wiped by ethanol after the test of each rat. The contralateral utilization ratio (%) = [(number of contralateral touches + ½ number of bilateral touches)/number of total touches] × 100%.

### Hematoxylin-eosin (HE) staining and Nissl staining

After the behavioral measurement, the rats in each group were euthanized, and the brain tissues of each group were harvested and fixed with paraformaldehyde. The brain tissues were hydrated with 70%, 80% and 95% gradient alcohol for 12 h each, and n-butyl alcohol for 6 h. The tissues were waxed at 60°C overnight, then were embedded and sliced continuously with the slice thickness of 6-8 μm. One slice was taken every 3 pieces.

HE staining: Paraffin sections were routinely washed by distilled water, stained with Mayer hematoxylin at room temperature for 5 min and flushed with tap water for l min to make the nucleus return to blue. Next, the sections were counterstained with 1% eosin alcohol solution for l min, followed by alcohol dehydration, xylene permeabilization and sealing. The nucleus was blue and the cytoplasm was purplish red.

Nissl staining: The coronal sections of the brain were attached to the slides, dried naturally, and then soaked in xylene for 10 min, 100% ethanol for 10 min, 95% ethanol for 5 min and 70% ethanol for 10 min. The sections were washed with distilled water and stained in 0.5% coke violet solution (pH = 3.9) for 30 min, then rinsed with distilled water for 3 min. Subsequently, the sections were decolorized in 70% ethanol, 95% ethanol (pH = 4.1) and 100% ethanol, followed by xylene permeabilization and sealing with neutral balsam. Nissl staining positive neurons were counted under a microscope. The staining standards were as follows: The positive neurons had clear nucleolus and blue cytoplasm, and the nuclei were thinly stained. Three sections in each case were selected and survival neurons (Nissl positive neurons) in 4 fields of view in each section were counted.

### High performance liquid chromatography (HPLC)

After the behavioral measurement, the rats in each group were euthanized, and the brain tissues of each group were weighed and homogenated with ultrasound. The supernatant of brain homogenate was collected after centrifugation at 4°C. The liquid collected after filtration with a 0.22 μm microporous filter was the sample solution to be tested. The chromatographic conditions were set as follows: chromatographic column: YWG-C18 column (250 mm × 46 mm); mobile phase A: domestic chromatographic pure methanol; mobile phase B: 0.1 mol/L sodium acetate, ultra-pure water configuration. The mobile phase A and B was mixed in a certain proportion and filtered with 0.22 μm microporous membrane. The sample quantity was 40 μL/time by ultrasonic degassing for 20 min, and then gradient elution was carried out. The mixed standard solution of different concentrations was used (40 μL each). The regression equation and linear range of the neurotransmitter dopamine were calculated with the peak area Y as the vertical coordinate and the sample concentration X (ng/mL) as the transverse coordinate. According to the measured peak area, the concentration of neurotransmitter dopamine in the sample solution was calculated according to the regression equation.

### Immunohistochemical staining

The sections were dewaxed, hydrated, and treated with 3% methanol-hydrogen peroxide solution for 15 min at 37°C, and added with normal goat serum sealant for 30 min at 37°C following antigen retrieval in sodium citrate buffer. Afterwards, the primary antibody to tyrosine hydroxylase (TH, 1 : 1000) was supplemented and the sections were washed several times, and then the sections were appended with horseradish peroxidase-labeled secondary antibody immunoglobulin G (IgG, 1 : 200) and washed several times. Lastly, the sections were developed with diaminobenzidine (DAB), followed by hematoxylin counterstaining, dehydration, permeabilization and neutral balsam sealing. TH immunoreactive neurons were observed and counted under a light microscope.

### Detection of superoxide dismutase (SOD), glutathione peroxidase (GSH-Px) and malondialdehyde (MDA) in striatum

The rats were euthanized to obtain the striatum of the substantia nigra. The brain tissues were weighed and homogenized by ultrasonic. The brain tissue homogenate was centrifuged at 4°C and the supernatant was collected. The protein concentration was determined by Coomassie brilliant blue method. The absorbance of MDA, SOD and GSH-Px in homogenate was measured at 532 nm, 550 nm and 422 nm respectively with a spectrophotometer based on the instructions of MDA, SOD and GSH-Px kits (NanJing JianCheng Bioengineering Institute, Nanjing, China).

### Enzyme-linked immunosorbent assay (ELISA)

The nigral tissues retained in the liquid nitrogen tank were taken out and maintained at the low temperature. Next, the samples in each group were diluted according to a fixed proportion of normal saline. After that, the homogenized samples were centrifuged at 4°C for 20 min (3000 rpm). The expression of tumor necrosis factor-α (TNF-α), interleukin-1β (IL-1β) and interleukin-6 (IL-6) in the supernatant of substantia nigra was detected by ELISA kits. The optical density (OD) value was measured at 450 nm (within 10 min after termination). All OD values should be calculated after zero-well value was deducted. The standard curve was drawn by computer Curve Expert 1. 3 analysis software, and the sample content was calculated.

### Reverse transcription quantitative polymerase chain reaction (RT-qPCR)

The Trizol method (Takara, Dalian, China) was implemented to extract the total RNA. Nanodrop2000 (Thermo Fisher Scientific, San Jose, CA, USA) was used to determine the concentration and quality of RNA. The complementary DNA (cDNA) was obtained according to the instructions of the reverse transcription kits (DRR047S, Takara, Dalian, China). The obtained cDNA was added with 65 μL diethyl pyrocarbonate (DEPC) and mixed. PCR primer was designed and synthesized by Shenzhen BGI (Shenzhen, China) (Table 1). U6 was taken as the internal reference of miR-375, and glyceraldehyde phosphate dehydrogenase (GAPDH) was taken as the internal reference of SP1, TNF-α, IL-6 and IL-1β. There were six duplicates in each gene of each sample. The 2^-ΔΔCt^ method [42] was used to analyze the ratio relation of target gene expression between the experimental group and the control group: ΔΔCt = [Ct _(target gene)_ - Ct _(internal control gene)_] _experimental group_ - [Ct _(target gene)_ - Ct _(internal control gene)_] _control group_.

**Table 1:** Primer sequence.

**Gene**	**Sequence (5′ - 3′)**
miR-375	Forward: 5′- CCCCAAGGCTGATGCTGAGAAG-3′
	Reverse: 5′- GCCGCCCGGCCCCGGGTCTTC-3′
U6	Forward: 5′- CTCGCTTCGCAGCACA -3′
	Reverse: 5′- AACGCTTCACGAATTTGCGT -3′
SP1	Forward: 5′-TGCAGCAGAATTGAGTCACC-3′
	Reverse: 5′-CACAACATACTGCCCACCAG-3′
TNF-α	Forward: 5′-TCAGCCGATTTGCCATTTCAT-3′
	Reverse: 5′-ACACGCCAGTCGCTTCACAGA-3′
IL-1β	Forward: 5′-GTCCTTTCACTTGCCCTCAT-3′
	Reverse: 5′-CAAACTGGTCACAGCTTTCGA-3′
IL-6	Forward: 5′-AAATGCCTCGTGCTGTCTGACC-3′
	Reverse: 5′-GGTGGGTGTGCCGTCTTTCATC-3′
GAPDH	Forward: 5′-AACGGATTTGGTCGTATTGGG-3′
	Reverse: 5′-TCGCTCCTGGAAGATGGTGAT-3′

Note: miR-375, microRNA-375; SP1, specificity protein 1; GAPDH, glyceraldehyde phosphate dehydrogenase.

### Western blot analysis

The proteins from cells were extracted and the protein concentrations were determined referring to the instructions of the bicinchoninic acid assay kits (Beyotime Institute of Biotechnology, Shanghai, China). The proteins were separated by 10% sodium dodecyl sulfate polyacrylamide gel electrophoresis and transferred to a nitrocellulose membrane. Subsequently, the protein samples were transferred to polyvinylidene fluoride membranes and blocked with 5% bovine serum albumin at 4°C. Afterwards, the membranes were added with the primary antibodies to SP1 (1 : 500), Caspase-3 (1 : 500), Bax (1 : 1000), Bcl-2 (1 : 500) (Abcam, Cambridge, MA, USA) and GAPDH (1: 1000) (Millipore Corporation, Bedford, CA, USA) and incubated at 4°C overnight. The membranes were then rinsed with Tris-buffered saline and Tween 3 times. The membranes were incubated with corresponding horseradish peroxidase-labeled secondary antibodies (Abcam, Cambridge, MA, USA) for 1 h at 37°C. An enhanced chemiluminescent solution was used for developing. The gray value of the target band was analyzed by Image J software.

### Dual luciferase reporter gene assay

A bioinformatics software http://www.targetscan.org was used to predict the targeting relationship between miR-375 and SP1 and the binding sites of miR-375 on SP1 3′-untranslated region (3’UTR). The restriction endonuclease Hind III and Spe I were introduced into the forward and reverse primers and the mutation sequence of the binding site was designed. The target sequence was synthesized by GenScript (Nanjing) Co., Ltd. (Nanjing, China). The target product and pMIR-REPORT™ Luciferase vector plasmid were digested by restriction enzyme Hind III and Spe I. The products were recycled and ligated with T4 DNA ligase, and transformed into Escherichia coli DH5α competent cells. In the 12-well plate, 1 × 10^5^ 293T cells were seeded into each well. The cells were cotransfected with recombinant plasmid and miR-375 mimics for 48 h according to the corresponding set group. After 48 h, the cell culture medium was discarded and phosphate buffered saline was added for 3-time washing. The luciferase activity was detected with a luciferase detection kit.

### Statistical analysis

All statistical analyses were performed using the SPSS 21.0 software (IBM SPSS, Inc., Chicago, IL, USA). The data in normal distribution were verified by Kolmogorov-Smirnov test. The measurement data were expressed as mean ± standard deviation. The t test was used for the comparison between the two groups, and one-way analysis of variance (ANOVA) was used for the comparison among multiple groups. After ANOVA analysis, the Tukey’s post-hoc test was used for pairwise comparison. *P* values ≤ 0.05 were considered statistically significant.

### Ethical statement

The experiment was approved by Guizhou provincial people’s hospital.

## References

[1] Vernier P, Moret F, Callier S, Snapyan M, Wersinger C, Sidhu A. The degeneration of dopamine neurons in Parkinson’s disease: insights from embryology and evolution of the mesostriatocortical system. Ann N Y Acad Sci. 2004; 1035:231–49. 10.1196/annals.1332.015 15681811

[2] Chan CS, Gertler TS, Surmeier DJ. A molecular basis for the increased vulnerability of substantia nigra dopamine neurons in aging and Parkinson’s disease. Mov Disord. 2010 (Suppl 1); 25:S63–70. 10.1002/mds.22801 20187241

[3] Xiong N, Huang J, Chen C, Zhao Y, Zhang Z, Jia M, Zhang Z, Hou L, Yang H, Cao X, Liang Z, Zhang Y, Sun S, et al. Dl-3-n-butylphthalide, a natural antioxidant, protects dopamine neurons in rotenone models for Parkinson’s disease. Neurobiol Aging. 2012; 33:1777–91. 10.1016/j.neurobiolaging.2011.03.007 21524431

[4] Hirsch EC, Höglinger G, Rousselet E, Breidert T, Parain K, Feger J, Ruberg M, Prigent A, Cohen-Salmon C, Launay JM. Animal models of Parkinson’s disease in rodents induced by toxins: an update. J Neural Transm Suppl. 2003; 65:89–100. 10.1007/978-3-7091-0643-3_6 12946051

[5] Ebert AD, Hann HJ, Bohn MC. Progressive degeneration of dopamine neurons in 6-hydroxydopamine rat model of Parkinson’s disease does not involve activation of caspase-9 and caspase-3. J Neurosci Res. 2008; 86:317–25. 10.1002/jnr.21480 17787016

[6] Lu J, Xu Y, Quan Z, Chen Z, Sun Z, Qing H. Dysregulated microRNAs in neural system: implication in pathogenesis and biomarker development in Parkinson’s disease. Neuroscience. 2017; 365:70–82. 10.1016/j.neuroscience.2017.09.033 28964753

[7] Wang Y, Yang Z, Le W. Tiny But Mighty: Promising Roles of MicroRNAs in the Diagnosis and Treatment of Parkinson’s Disease. Neurosci Bull. 2017; 33:543–51. 10.1007/s12264-017-0160-z 28762215 PMC5636733

[8] Harraz MM, Dawson TM, Dawson VL. MicroRNAs in Parkinson’s disease. J Chem Neuroanat. 2011; 42:127–30. 10.1016/j.jchemneu.2011.01.005 21295133 PMC3163813

[9] Kabaria S, Choi DC, Chaudhuri AD, Mouradian MM, Junn E. Inhibition of miR-34b and miR-34c enhances α-synuclein expression in Parkinson’s disease. FEBS Lett. 2015; 589:319–25. 10.1016/j.febslet.2014.12.014 25541488 PMC4306645

[10] Miñones-Moyano E, Porta S, Escaramís G, Rabionet R, Iraola S, Kagerbauer B, Espinosa-Parrilla Y, Ferrer I, Estivill X, Martí E. MicroRNA profiling of Parkinson’s disease brains identifies early downregulation of miR-34b/c which modulate mitochondrial function. Hum Mol Genet. 2011; 20:3067–78. 10.1093/hmg/ddr210 21558425

[11] Kim J, Inoue K, Ishii J, Vanti WB, Voronov SV, Murchison E, Hannon G, Abeliovich A. A MicroRNA feedback circuit in midbrain dopamine neurons. Science. 2007; 317:1220–24. 10.1126/science.1140481 17761882 PMC2782470

[12] Cho HJ, Liu G, Jin SM, Parisiadou L, Xie C, Yu J, Sun L, Ma B, Ding J, Vancraenenbroeck R, Lobbestael E, Baekelandt V, Taymans JM, et al. MicroRNA-205 regulates the expression of Parkinson’s disease-related leucine-rich repeat kinase 2 protein. Hum Mol Genet. 2013; 22:608–20. 10.1093/hmg/dds470 23125283 PMC3542867

[13] Poy MN, Eliasson L, Krutzfeldt J, Kuwajima S, Ma X, Macdonald PE, Pfeffer S, Tuschl T, Rajewsky N, Rorsman P, Stoffel M. A pancreatic islet-specific microRNA regulates insulin secretion. Nature. 2004; 432:226–30. 10.1038/nature03076 15538371

[14] Abdelmohsen K, Hutchison ER, Lee EK, Kuwano Y, Kim MM, Masuda K, Srikantan S, Subaran SS, Marasa BS, Mattson MP, Gorospe M. miR-375 inhibits differentiation of neurites by lowering HuD levels. Mol Cell Biol. 2010; 30:4197–210. 10.1128/MCB.00316-10 20584986 PMC2937556

[15] Li H, Tao R, Wang J, Xia L. Upregulation of miR-375 level ameliorates morphine analgesic tolerance in mouse dorsal root ganglia by inhibiting the JAK2/STAT3 pathway. J Pain Res. 2017; 10:1279–87. 10.2147/JPR.S125264 28603428 PMC5457281

[16] Citron BA, Dennis JS, Zeitlin RS, Echeverria V. Transcription factor Sp1 dysregulation in Alzheimer’s disease. J Neurosci Res. 2008; 86:2499–504. 10.1002/jnr.21695 18449948

[17] Citron BA, Saykally JN, Cao C, Dennis JS, Runfeldt M, Arendash GW. Transcription factor Sp1 inhibition, memory, and cytokines in a mouse model of Alzheimer’s disease. Am J Neurodegener Dis. 2015; 4:40–48. 26807343 PMC4700125

[18] Chen T, Hou R, Li C, Wu C, Xu S. MPTP/MPP+ suppresses activation of protein C in Parkinson’s disease. J Alzheimers Dis. 2015; 43:133–42. 10.3233/JAD-140126 25061051

[19] Villa C, Ridolfi E, Fenoglio C, Ghezzi L, Vimercati R, Clerici F, Marcone A, Gallone S, Serpente M, Cantoni C, Bonsi R, Cioffi S, Cappa S, et al. Expression of the transcription factor Sp1 and its regulatory hsa-miR-29b in peripheral blood mononuclear cells from patients with Alzheimer’s disease. J Alzheimers Dis. 2013; 35:487–94. 10.3233/JAD-122263 23435408

[20] Ramanan VK, Saykin AJ. Pathways to neurodegeneration: mechanistic insights from GWAS in Alzheimer’s disease, Parkinson’s disease, and related disorders. Am J Neurodegener Dis. 2013; 2:145–75. 24093081 PMC3783830

[21] Wang F, Li Y, Zhou J, Xu J, Peng C, Ye F, Shen Y, Lu W, Wan X, Xie X. miR-375 is down-regulated in squamous cervical cancer and inhibits cell migration and invasion via targeting transcription factor SP1. Am J Pathol. 2011; 179:2580–88. 10.1016/j.ajpath.2011.07.037 21945323 PMC3204087

[22] Gai Y, Zhang J, Wei C, Cao W, Cui Y, Cui S. miR-375 negatively regulates the synthesis and secretion of catecholamines by targeting Sp1 in rat adrenal medulla. Am J Physiol Cell Physiol. 2017; 312:C663–72. 10.1152/ajpcell.00345.2016 28356269

[23] Titze-de-Almeida R, Titze-de-Almeida SS. miR-7 Replacement Therapy in Parkinson’s Disease. Curr Gene Ther. 2018; 18:143–53. 10.2174/1566523218666180430121323 29714132

[24] Zhang X, Yang R, Hu BL, Lu P, Zhou LL, He ZY, Wu HM, Zhu JH. Reduced Circulating Levels of miR-433 and miR-133b Are Potential Biomarkers for Parkinson’s Disease. Front Cell Neurosci. 2017; 11:170. 10.3389/fncel.2017.00170 28690499 PMC5481393

[25] He XX, Chang Y, Meng FY, Wang MY, Xie QH, Tang F, Li PY, Song YH, Lin JS. MicroRNA-375 targets AEG-1 in hepatocellular carcinoma and suppresses liver cancer cell growth in vitro and in vivo. Oncogene. 2012; 31:3357–69. 10.1038/onc.2011.500 22056881

[26] Ding L, Xu Y, Zhang W, Deng Y, Si M, Du Y, Yao H, Liu X, Ke Y, Si J, Zhou T. MiR-375 frequently downregulated in gastric cancer inhibits cell proliferation by targeting JAK2. Cell Res. 2010; 20:784–93. 10.1038/cr.2010.79 20548334

[27] Chang C, Shi H, Wang C, Wang J, Geng N, Jiang X, Wang X. Correlation of microRNA-375 downregulation with unfavorable clinical outcome of patients with glioma. Neurosci Lett. 2012; 531:204–08. 10.1016/j.neulet.2012.10.021 23103713

[28] Tsukamoto Y, Nakada C, Noguchi T, Tanigawa M, Nguyen LT, Uchida T, Hijiya N, Matsuura K, Fujioka T, Seto M, Moriyama M. MicroRNA-375 is downregulated in gastric carcinomas and regulates cell survival by targeting PDK1 and 14-3-3zeta. Cancer Res. 2010; 70:2339–49. 10.1158/0008-5472.CAN-09-2777 20215506

[29] Harris T, Jimenez L, Kawachi N, Fan JB, Chen J, Belbin T, Ramnauth A, Loudig O, Keller CE, Smith R, Prystowsky MB, Schlecht NF, Segall JE, Childs G. Low-level expression of miR-375 correlates with poor outcome and metastasis while altering the invasive properties of head and neck squamous cell carcinomas. Am J Pathol. 2012; 180:917–28. 10.1016/j.ajpath.2011.12.004 22234174 PMC3349885

[30] Bhinge A, Namboori SC, Bithell A, Soldati C, Buckley NJ, Stanton LW. MiR-375 is Essential for Human Spinal Motor Neuron Development and May Be Involved in Motor Neuron Degeneration. Stem Cells. 2016; 34:124–34. 10.1002/stem.2233 26507573

[31] Zhao X, Shu F, Wang X, Wang F, Wu L, Li L, Lv H. Inhibition of microRNA-375 ameliorated ketamine-induced neurotoxicity in human embryonic stem cell derived neurons. Eur J Pharmacol. 2019; 844:56–64. 10.1016/j.ejphar.2018.11.035 30500346

[32] Reyes S, Fu Y, Double KL, Cottam V, Thompson LH, Kirik D, Paxinos G, Watson C, Cooper HM, Halliday GM. Trophic factors differentiate dopamine neurons vulnerable to Parkinson’s disease. Neurobiol Aging. 2013; 34:873–86. 10.1016/j.neurobiolaging.2012.07.019 22926168

[33] Ryu H, Lee J, Zaman K, Kubilis J, Ferrante RJ, Ross BD, Neve R, Ratan RR. Sp1 and Sp3 are oxidative stress-inducible, antideath transcription factors in cortical neurons. J Neurosci. 2003; 23:3597–606. 10.1523/JNEUROSCI.23-09-03597.2003 12736330 PMC6742168

[34] Xu Y, Porntadavity S, St Clair DK. Transcriptional regulation of the human manganese superoxide dismutase gene: the role of specificity protein 1 (Sp1) and activating protein-2 (AP-2). Biochem J. 2002; 362:401–12. 10.1042/bj3620401 11853549 PMC1222401

[35] García-Morales V, Rodríguez-Bey G, Gómez-Pérez L, Domínguez-Vías G, González-Forero D, Portillo F, Campos-Caro A, Gento-Caro Á, Issaoui N, Soler RM, Garcera A, Moreno-López B. Sp1-regulated expression of p11 contributes to motor neuron degeneration by membrane insertion of TASK1. Nat Commun. 2019; 10:3784. 10.1038/s41467-019-11637-4 31439839 PMC6706379

[36] Eacker SM, Dawson TM, Dawson VL. Understanding microRNAs in neurodegeneration. Nat Rev Neurosci. 2009; 10:837–41. 10.1038/nrn2726 19904280 PMC4120241

[37] Brodersen P, Voinnet O. Revisiting the principles of microRNA target recognition and mode of action. Nat Rev Mol Cell Biol. 2009; 10:141–48. 10.1038/nrm2619 19145236

[38] Ghildiyal M, Zamore PD. Small silencing RNAs: an expanding universe. Nat Rev Genet. 2009; 10:94–108. 10.1038/nrg2504 19148191 PMC2724769

[39] Cui F, Wang S, Lao I, Zhou C, Kong H, Bayaxi N, Li J, Chen Q, Zhu T, Zhu H. miR-375 inhibits the invasion and metastasis of colorectal cancer via targeting SP1 and regulating EMT-associated genes. Oncol Rep. 2016; 36:487–93. 10.3892/or.2016.4834 27222350

[40] Jungling A, Reglodi D, Karadi ZN, Horvath G, Farkas J, Gaszner B, Tamas A. Effects of Postnatal Enriched Environment in a Model of Parkinson’s Disease in Adult Rats. Int J Mol Sci. 2017; 18:E406. 10.3390/ijms18020406 28216584 PMC5343940

[41] Barros AS, Crispim RY, Cavalcanti JU, Souza RB, Lemos JC, Cristino Filho G, Bezerra MM, Pinheiro TF, de Vasconcelos SM, Macêdo DS, de Barros Viana GS, Aguiar LM. Impact of the Chronic Omega-3 Fatty Acids Supplementation in Hemiparkinsonism Model Induced by 6-Hydroxydopamine in Rats. Basic Clin Pharmacol Toxicol. 2017; 120:523–31. 10.1111/bcpt.12713 27883274

[42] Tuo YL, Li XM, Luo J. Long noncoding RNA UCA1 modulates breast cancer cell growth and apoptosis through decreasing tumor suppressive miR-143. Eur Rev Med Pharmacol Sci. 2015; 19:3403–11. 26439035

